# Analysis of risk factors for *T. brucei rhodesiense *sleeping sickness within villages in south-east Uganda

**DOI:** 10.1186/1471-2334-8-88

**Published:** 2008-06-30

**Authors:** Thomas Zoller, Eric M Fèvre, Susan C Welburn, Martin Odiit, Paul G Coleman

**Affiliations:** 1 Medizinische Klinik mit Schwerpunkt Infektiologie und Pneumologie, Charité – Universitätsmedizin Berlin, Charitéplatz 1, 10117 Berlin, Germany; 2 Centre for Infectious Diseases, University of Edinburgh, Ashworth Labs, Kings Buildings, West Mains Road, Edinburgh, EH9 3JT, UK; 3 Centre for Infectious Diseases, University of Edinburgh, Easter Bush, Roslin, Midlothian, Edinburgh EH25 9RG, UK; 4 UACP, PO Box 25589, Kampala, Uganda. Formerly, Sleeping Sickness Programme, LIRI Hospital, PO Box 96, Tororo, Uganda; 5 London School of Hygiene and Tropical Medicine, Disease Control and Vector Biology Unit, Keppel Street, London WC1E 7HT, UK

## Abstract

**Background:**

Sleeping sickness (HAT) caused by *T.b. rhodesiense *is a major veterinary and human public health problem in Uganda. Previous studies have investigated spatial risk factors for *T.b. rhodesiense *at large geographic scales, but none have properly investigated such risk factors at small scales, i.e. within affected villages. In the present work, we use a case-control methodology to analyse both behavioural and spatial risk factors for HAT in an endemic area.

**Methods:**

The present study investigates behavioural and occupational risk factors for infection with HAT within villages using a questionnaire-based case-control study conducted in 17 villages endemic for HAT in SE Uganda, and spatial risk factors in 4 high risk villages. For the spatial analysis, the location of homesteads with one or more cases of HAT up to three years prior to the beginning of the study was compared to all non-case homesteads. Analysing spatial associations with respect to irregularly shaped geographical objects required the development of a new approach to geographical analysis in combination with a logistic regression model.

**Results:**

The study was able to identify, among other behavioural risk factors, having a family member with a history of HAT (p = 0.001) as well as proximity of a homestead to a nearby wetland area (p < 0.001) as strong risk factors for infection. The novel method of analysing complex spatial interactions used in the study can be applied to a range of other diseases.

**Conclusion:**

Spatial risk factors for HAT are maintained across geographical scales; this consistency is useful in the design of decision support tools for intervention and prevention of the disease. Familial aggregation of cases was confirmed for *T. b. rhodesiense *HAT in the study and probably results from shared behavioural and spatial risk factors amongmembers of a household.

## Background

Human African trypanosomiasis (HAT) or sleeping sickness is a re-emerging disease which poses a major public health problem in certain regions of Africa. The disease occurs in a patchy distribution over 36 countries across sub-Saharan Africa. Although long-term asymptomatic carriers have been described [1] little is known about the natural course of infection; the disease is usually fatal in the absence of treatment. A breakdown in control measures in many areas has been followed by a resurgence of HAT since the 1970s [2,3]. An estimated 300.000 new human infections occur annually, causing an estimated 46.000 deaths per year [4,5]. A recent programme set up in the year 2000 including intensified surveillance and control measures, training of health personnel and access to drugs has made significant contributions to the recently observed decline in the number of newly reported cases, mainly for *T.b. gambiense *[5,6].

The infection occurs in two distinct forms: East-African (*T. b. rhodesiense*) and West-African trypanosomiasis (*T. b. gambiense*). Although human-infective parasites have also found in animals in West Africa [7], humans are thought to be the principal reservoir hosts for *T.b. gambiense *HAT. East-African HAT is a zoonosis [8] and transmission requires the presence of suitable reservoir animals (e.g. domestic livestock, wild bovids). Uganda is the only country where both forms of HAT are prevalent. *T. b. rhodesiense *HAT has been endemic in southern and eastern Uganda for more than 100 years, but outbreaks of HAT in areas previously free of the disease continue to occur [9-11]. A well documented outbreak in Soroti District, central Uganda, was linked to cattle movements from an endemic to a previously non-endemic area and proximity to a local cattle market was identified as a risk factor for infection [12].

The main determinants of transmission are the presence of the tsetse fly supported by appropriate habitat and the presence of the mammalian reservoir host, which is, in south-eastern Uganda, principally cattle. Anthropogenic factors such as the location of villages, the main occupation of villagers (e.g. subsistence agriculture) and certain activities like fishing, washing clothes and contact with cattle are also thought to influence HAT transmission, though they have rarely been quantified. These factors ultimately determine the intensity of contact between the fly, reservoir animals and humans.

In Eastern Uganda, HAT is transmitted by *Glossina fuscipes fuscipes*, a strongly zoophilic tsetse species, taking only a small percentage of bloodmeals (about 16–23%) on humans [13]. Similarly to *G. palpalis *in West Africa, the typical habitat of *G. f. fuscipes *is bushes and thickets around open water (e.g. riverine vegetation, wells, ponds and swamps). In Eastern Uganda, a district-level analysis found that proximity of villages to long vegetation swamp habitat was a risk factor for the presence of sleeping sickness in a village [14]. In the study area, cattle are one of the most important economic possessions for village inhabitants. Cattle are kept in numbers of 1–5 cows per family, either grazing around the homestead, or in communal herds of around 50–100 animals on grazing sites within ornear the village borders.

In the present work, we investigate whether spatial risk factorsidentified at regional scales are maintained at smaller geographical areas (the within-village scale). Whereas there have been previous studies at a number of spatial scales linking landscape features to risk of human and animal trypanosomiasis transmission [14-16], risk factors at the within-village scale have never been adequately investigated. In addition, this study uses a case-control methodology to combine an analysis of behavioural risk factors with a GIS-supported analysis of the spatial risk factors.

## Methods

The study was undertaken in Tororo District, south-east Uganda (longitude 33.8–34.0; latitude 0.5–0.9). The study area and the spatial distribution of all villages in the District, have been previously described in detail elsewhere [14,17]. Study villages were selected on the basis of reported sleeping sickness cases at the Livestock Health Research Institute (LIRI) sleeping sickness hospital in Tororo. With regard to accuracy of information in the patient records and to ensure that changes in village structure and land use over time did not influence the results of the study, all cases within a period of three years before the start of the study were included in the database. Cases in the records had originally been identified through passive case detection using clinical examination and microscopic detection of trypanosomes in blood and CSF [8]. Tororo District is located in south-eastern Uganda close to the Kenyan border and has a surface area of 1175 km^2 ^and around 400.000 inhabitants. Over 90% of the population lives in rural areas, mostly villages with up to 1000 inhabitants per village. Almost all of the village inhabitants of Tororo district are subsistence farmers and most of the agricultural land in the area is used for subsistence farming. All villages included in this study are located in the catchment area of LIRI hospital and within a distance of 10 km of the town of Tororo. Most villages in this area have a similar structure; village borders are often defined by natural barriers, mostly small rivers and wetlands. Boreholes may be scattered through the village but open water sites tend to be on the village boundaries. Cattle are grazed extensively and follow daily routes from pasture to watering sites. The area between the grazing sites and the outermost homesteads of the village is occupied by fields and gardens; homesteads themselves are generally many hundreds of metres from the physical boundaries of the village.

All participants provided witnessed oral informed consent before the interview (if a participant was a minor, permission was sought from the parents) and the study was approved by the Tororo District administration, and the respective village chairperson. The study received ethical clearance from the ethical committee of the London School of Hygiene and Tropical Medicine (UK).

### Behavioural, environmental and socio-demographic risk factors

To study behavioural and socio-demographic risk factors for infection with *T.b. rhodesiense *as well as to evaluate the role of land use around a homestead in HAT risk, a matched case-control study was conducted. 17 villages with at least one case over the period of three years were included in the study. A total of 75 cases of HAT were identified from treatment records of LIRI hospital and visited. Controls with no clinical signs or history of HAT infection were selected from the same villages and matched to cases by age-group (± 5 years, minimum age = 10 years) and sex. One control was chosen for each case, and controls were identified by randomly selecting a household from a list of households in a village. If no suitable age- and sex-matched control was available for an interview in the selected household, the next neighbouring households were visited until a suitable control who agreed to participate was identified.

After obtaining oral consent, cases and controls were interviewed using a standardised questionnaire. The data were entered into EpiInfo6 and transferred to STATA8 (STATA 8, College Station, TX, USA) for further statistical analysis. The dataset contained categorical (e.g. presence of cattle around the homestead) as well as continuous (e.g. number of cattle present around homestead) variables. Conditional logistic regression was used to calculate odds ratios to estimate the risk of HAT in both a univariate and multivariate analysis. For the latter, variables were selected using a backward stepwise selection procedure, retaining only variables with a p < 0.1 for the final model.

### Spatial risk factors

In order to conduct a detailed analysis of spatial risk factors at the household level, we selected the four villages reporting the highest number of cases (6–10 cases per village). All homesteads in these villages were georeferenced – the locations of all homesteads in each village were mapped using a hand-held Global Positioning System (GPS; Garmin, Olathe, KS, USA). Geographical features of interest including wetland boundaries were also mapped using a combination of data extraction from digitised versions of recently updated 1:50,000 topographical maps (available from the Department of Maps and Surveys, Entebbe, Uganda), and field mapping. Maps were digitised in the ArcView 3.2 Geographical Information System (GIS; ESRI, Redlands, USA), using the Image Warp extension [18]. Homesteads were assigned a status of case or non-case depending on the presence of sleeping sickness in a member of the home in the previous 3 years.

Wetlands as objects for geographical analysis were irregularly shaped; such features present methodological problems in spatial analysis (e.g. taking the shortest distance of a homestead to the nearest border of a wetland alone does not fully reflect the spatial complexity in a branched wetland system). The spatial relationship between the homestead and the wetland was represented by drawing buffer zones of variable sizes around all homesteads of a village; buffers with a radius ranging from 400 m up to 5000 m were used, measuring, as an output, the proportion of the landscape surrounding each homestead that belonged to the wetland class. The buffer size was based on knowledge of the village layouts and the range of daily activity patterns of the inhabitants of the villages. Positive case status in homesteads was defined as a categorical outcome variable and the intersecting proportion of buffers around homesteads with the wetland as a continuous exposure variable. Logistic regression was used to detect an association of positive case status of a homestead and intersecting proportion of buffers. Since all four villages were analysed in a common dataset, the final model included village clustering of cases as a possible confounding factor.

## Results

### Study population

The study population consisted of 75 case/control pairs coming from 17 different villages in Tororo district. The mean age of the study population was 37.2 years (± SD 19.6 years, minimum age: 10 years, maximum age 81 years). 62% of participants were male and 65% of participants were married (for details see Table 1). The study population was very homogeneous; most of participants declared subsistence farming as the main occupation and most participants were livestock owners (mainly cows, goats and chickens). In the study population, we observed relevant and significant differences between women and men with regard to occupation and activities involving potential risks for HAT infection (e.g. herding cattle, collecting water or firewood, for details see Table 2). Most of the village dwellers that left the village regularly were children visiting school.

**Table 1:** Socio-demographic characteristics of the case-control study population

Age	• Mean age: 37.2 years (± SD 19.6 years)							
	• Minimum age: 10 years							
	• Maximum age 81 years							
Age distribution	10–19	20–29	30–39	40–49	50–59	60–69	70–79	80–89
	27	34	18	15	27	19	4	3
Gender	• 62% male							
	• 38% female							
Marital status	• 65% married,							
	• 35% single, widowed or divorced							
Occupation and source of income	• 95% agriculture							
	• 93% working in household							
	• 91% livestock ownership (personal or family)							
Cattle	• 63% of study population owned cattle (personal or family).							
	• A mean of 3.4 cows were owned by one family (± SD 3.4), ranging from 1 to a maximum of 22 cows.							

**Table 2:** Occupational characteristics of the study population

	Total (n = 150)	Men (n = 93)	Women (n = 57)	p*
Herding cattle	43%	54%	26%	0.001
Visiting cattle market	55%	57%	52%	0.602
Collecting water	85%	79%	95%	0.007
Collecting firewood	67%	56%	86%	<0.001
Washing clothes outside homestead	41%	35%	51%	0.063

*) χ^2 ^test for difference in activity between men and women

### Behavioural, environmental and socio-demographic risk factors

On a within-village scale, we investigated several socio-demographic and behavioural risk factors for infection with HAT as well as a possible association of HAT risk with the type of land use around homesteads. With regard to behaviour and occupation, age and sex were considered the two most important confounders and were eliminated by matching of cases and controls at the design stage of the study. Univariate results are reported in Table 3; in the univariate analysis, having a family member who previously had sleeping sickness (OR 3.6, CI_95 _1.34–9.7, p = 0.011) and the presence of cassava as a crop type around the homestead (OR 1.93, CI_95 _1.01–3.68, p = 0.046) were found to be significantly associated with sleeping sickness. Other risk factors showed either no or only a weak association with the outcome and none of them reached statistical significance. Visiting the cattle market, involving regular absence from the village, seemed to be negatively associated with HAT (borderline statistical significance, OR 0.48, CI95 0.22–1.01, p = 0.053).

**Table 3:** Results of univariate analysis

		OR	CI_95_	p	
Land use around homestead	Cassava	1.93	1.01–3.68	0.046	*
	Bananas	0.47	0.2–1.09	0.079	
	Millet	0.9	0.39–2.14	0.827	
	Maize	0.94	0.46–1.9	0.857	
	Cattle	1.46	0.72–2.96	0.292	
	No. of cattle	1.01	0.88–1.17	0.833	+
	Pigs	0.78	0.29–2.09	0.618	
Familial characteristics	Sleeping sickness in member of family	3.6	1.34–9.7	0.011	*
	Family members in neighbouring villages	0.63	0.21–1.91	0.41	
	Marital status	1.42	0.54–3.75	0.469	++
Livestock ownership	Livestock ownership	0.67	0.19–2.36	0.530	
	Cattle	1.53	0.77–3.1	0.227	
	No. of cattle owned	1	0.89–1.1	0.829	+
	Chicken	0.75	0.38–1.46	0.400	
	Pigs	0.67	0.32–1.38	0.435	
	Goats	1	0.5–2	1	
	Dogs	0.93	0.44–1.98	0.847	
Occupation	Agriculture	0.17	0.02–1.38	0.097	
	Working in Household	1.25	0.34–7.65	0.739	
	Livestock (source of income)	0.86	0.289–2.55	0.782	
	Regular work outside village	0.5	0.19–1.33	0.166	
	Herding cattle	0.8	0.37–1.7	0.565	
	Visiting cattle market	0.48	0.22–1.01	0.053	
	Collecting water	0.9	0.39–2.14	0.827	
	Collecting firewood	0.93	0.44–1.98	0.847	
	Washing clothes outside homestead	1.25	0.65–2.41	0.506	
	No. of times clothes washed outside homestead (week)	1.24	0.83–1.82	0.293	+

*) Significant at 95% level (p < 0.05)

+) Continuous variable

++) 1 = married, 0 = single, divorced or widowed

A multivariate model was fitted to the data including those variables retained in a stepwise selection procedure (see above); the remaining variables in the model all appeared to be risk factors of potential relevance for HAT. In the multivariate analysis (see Table 4), again the strongest predictor for infection with HAT was a family member with history of HAT (OR 16.23, CI_95 _2.97–88.74, p = 0.001; Table 3). Also, villagers who stated that their main occupation was working in the household (in contrast to working in fields or gardens) appeared to have a higher risk of contracting HAT (OR 12.52, CI_95 _1.48–106.4, p = 0.02). Those village inhabitants who left the village regularly for work had a lower risk of contracting HAT (OR 0.14, CI_95 _0.03–0.73, p = 0.02), very similar to those who stated having family members in neighbouring villages (OR 0.12, CI95 0.02–0.72, p = 0.02), indicating regular travel outside the village.

**Table 4:** Results of multivariate analysis

		OR	CI_95_	p
Land use around homestead	Cassava	2.24	0.95–5.21	0.062
	Bananas	0.2	0.06–0.75	0.017
	Cattle	3.08	1.16–8.14	0.023
Familial characteristics	Sleeping sickness in member of family	16.23	2.97–88.74	0.001
	Family members in neighbouring villages	0.12	0.02–0.72	0.02
	Dogs	0.23	0.06–0.91	0.037
Occupation	Agriculture	0.1	0.01–1.47	0.094
	Working in Household	12.52	1.48–106.04	0.02
	Regular work outside village	0.14	0.03–0.73	0.02

With regard to land use and the presence of reservoir animals around the homestead, the presence of cattle seemed to increase the risk of contracting HAT (OR 3.08, CI_95 _1.16–8.14, p = 0.023). Unexpectedly, we observed a protective effect of banana plants around the homestead (OR 0.2, CI_95 _0.06–0.75, p = 0.017) and an increased, albeit not significantly increased risk for HAT when cassava was cultivated around the homestead (OR 2.24, CI_95 _0.95–5.21, p = 0.062). Possessing dogs was negatively correlated with the risk of contracting HAT (OR 0.23, CI_95 _0.06–0.91, p = 0.037).

### Spatial risk factors

**Figure 1:**
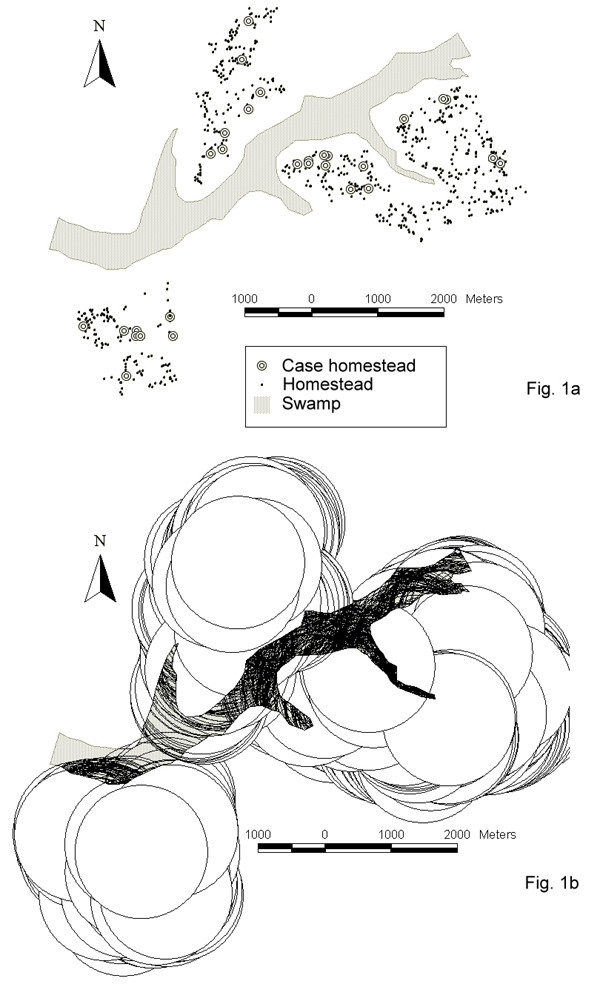
Fig. 1a shows the location of four villages around the central wetland. Homesteads where a case of sleeping sickness occurred during the past 3 years are marked with an open circle. Fig. 1b shows an example of buffers (radius 1000 m) drawn around homesteads to assess proximity of a homestead to the wetland.

Figure 1a gives an overview of the study area. The four study villages included in the spatial analysis are situated around a central wetland zone. As demonstrated on the map, homesteads with cases of HAT tended to be located more closely to the wetland than other homesteads of the same village. To quantify the relationship between the occurrence of HAT in a homestead and distance to the irregularly shaped wetland, buffers were drawn around each homestead. Figure 1b shows an example of buffers used to assess proximity of a homestead to the wetland, where proximity is expressed as the proportion of the buffer intersecting with the wetland area polygon.

**Figure 2:**
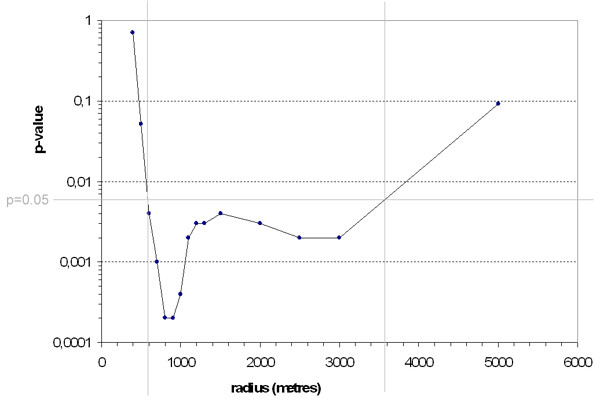
Result of logistic regression analysis for association of sleeping sickness with intersecting proportions of buffers with wetland area. P-values plotted as a function of buffer radius.

Figure 2 shows the results of the logistic regression analysing the correlation of positive case status of a homestead with the proportion of the intersecting area of the respective buffer with the wetland. Significance levels (p-value) of the correlation are plotted against buffer size. Above a radius of 500 m, a significant correlation of positive case status with buffer size covering wetland is observed (p < 0.05). This association is most significant (p < 0.001) at a buffer radius from 800 m to 900 m and remains significant up to a radius of 3000 m (p < 0.05). With increasing buffer size above 3000 m, all buffers include a large area of the wetland and differences between case- and non-case homesteads diminish to non-significance.

## Discussion

HAT transmission patterns are complex and, as outlined above, depend on a number of human, reservoir and vector related factors. HAT typically affects individuals scattered over a village and the total number of reported cases available for study is limited; in this area of Uganda, under-reporting of cases is a well-recognised problem [19]. Investigations of risk factors for infection with HAT have therefore been limited. In the present study of within-village risk factors, we used a combined approach including a behavioural risk factor analysis as well as a spatial risk factor analysis.

Behavioural risk factors (e.g. fishing, collecting firewood or water, cattle herding) have been thought to be of major importance for contracting HAT [20-22]. In our case-control study, having a family member with history of HAT was the strongest risk factor positively associated with HAT infection, followed by the need for regularly leaving the village for work outside the village (negative association). Both results are in line with the only comparable study available which was carried out in the Busoga region (neighbouring Tororo district) by Okia *et al*. in 1994 [22], where a similar correlation was found. Khonde *et al*. [23] reported familial aggregation among mothers and their children and between siblings, in a *T.b. gambiense *focus in Democratic Republic of Congo. Several studies have now reported this phenomenon in HAT; it could be a result of either common exposure to vectors or common contact with reservoir animals. Localised transmission around a family home is less likely due to the ecology of the vector, which typically prefers bushes and thickets around wetlands or rivers, away from homesteads; data on breeding sites and of movement activity of tsetse flies in this area illustrates that these riverine flies only travel around 350 metres [24]. There is a possibility that familial aggregation of cases could be the result of shared genetic susceptibility to infection with *T.b. rhodesiense *[25]; this hypothesis could however not be further elucidated by this study. The results presented here may however be a basis for further investigation of common genetic patterns increasing susceptibility to HAT in the study area.

A second finding is that village inhabitants who spend a significant amount of time outside the village seem to have a lower risk for HAT infection (regular work outside village, having family members in neighbouring villages, visiting cattle market (borderline significance)) whereas activities involving presence in the village during the entire day (working in household as main occupation) appear to carry a higher risk of HAT. These results were unexpected, since Okia *et al*. describe a higher risk for village inhabitants regularly leaving the village or collecting firewood in a study in Busoga [22].

Water contact activities such as washing clothes or fetching water were believed to carry a high risk for HAT infection due to the proximity to the vector habitat. This study however found no direct association between these activities and HAT infection.

There was a male predominance in all cases of HAT in this study (63%) and daily activities potentially carrying a risk for HAT infection showed a gender-dependent distribution. Those activities with most significant gender differences all turned out to be not significant predictors for HAT risk. The only activity for which we observed a near equal gender distribution was visiting the cattle market, which, in turn, was a risk factor of borderline statistical significance in the case-control study. This suggests that the observed male predominance in the case records of LIRI does not imply a higher risk for men to contract HAT in this area *per se*.

Our results indicate that in this HAT focus, landuse around a homestead may play a role in the risk of HAT for village inhabitants. The positive risk of presence of cattle around homesteads with HAT in the homestead suggests that human-livestock interaction impacts on transmission risk. In Tororo district, 3–7% of cattle have been found to carry infections with *T. b. rhodesiense *[26,27], and elsewhere, this figure is up to 18% [28]. The association of cassava with HAT infection and a possible protective effect of banana trees was unexpected; a brief report describes coffee, banana trees and *Lantana camara *thickets as habitats of *G. fuscipes fuscipes *[29]. This observation needs to be further clarified by further research and to be complemented by entomological studies.

Studies of environmental variables as risk factors or predictors of sleeping sickness have been carried out at a range of spatial scales. Robinson *et al*. [16] have investigated low resolution data in relation to tsetse habitat suitability, and Hendrickx *et al*. [30] investigated animal trypanosomiasis occurrence in relation to agricultural and herd-related macro-scale variables in Togo. In Uganda, Odiit *et al*. [14] used 30-metre resolution satellite imagery and found that proximity of village centroids to a long vegetation swamp class was a risk factor for presence of cases in a village. The distribution of early and late stage HAT cases around health centres in south-east Uganda was also studied by *Odiit et al*. [17]. To complement previous studies carried out at larger spatial scales, in this study we conducted an analysis of within-village spatial risk factors. We found that the proximity to wetland as significant risk factor for HAT is maintained as a consistent feature of HAT epidemiology even within villages, suggesting that it is robust indicator of risk.

Having a family member with HAT, the strongest risk factor for HAT in our study, might also be an effect of sharing common spatial risk factors; those families whose homestead is located near a swamp will have more family members at risk than families living in other parts of the village.

In the light of other risk factors with a spatial component from our case-control study (regular work outside village and family members in neighbouring villages), this study underlines the importance of spatial parameters in assessing the risk for HAT in this area of south-eastern Uganda and emphasises that behavioural risk factors alone may not be sufficient to identify individuals at high risk for contracting HAT. This research adds to the body of knowledge required for the design of decision support tools for *T.b. rhodesiense *HAT control; further research is, however, needed to complement the data in this report, including up-to-date entomological data.

Finally, demonstrating spatial associations with an irregularly shaped area (wetland) posed considerable methodological problems as these associations can be overrepresented in the entire dataset when a group of homesteads is in proximity to a narrow branch of a wetland. The method we use to analyse data from buffers around defined points eliminates this type of error. This method may be applied to the study complex spatial associations in a range of other diseases.

## Conclusion

The analysis of both spatial and behavioural risk factors has provided within-village data on HAT risk that can be used as a basis for designing further studies as well as for developing control and intervention programmes in this HAT focus. *T.b. rhodesiense *HAT has previously been shown to be associated with certain landscape features at larger geographical scales; that these relationships are maintained at smaller scales (which are also those most relevant to the individual's risk of exposure) emphasises their relevance for control. The results presented will be useful for the design of specific, targeted control interventions and the implementation of preventive measures (e.g. in land-use planning) in order to reduce the risk of HAT for village inhabitants in south-eastern Uganda. We recommend studies at an equivalent scale in other *T.b. rhodesiense *foci to better understand risk factors and to improve HAT management elsewhere.

## Competing interests

The authors declare that they have no competing interests.

## Authors' contributions

All authors conceived of the study. TZ, EMF, PGC and MO designed the data collection, TZ and MO conducted the fieldwork, and TZ, EMF and PG analysed the data. TZ, EMF and PGC drafted the manuscript. All authors have read and approved the final manuscript.

## Pre-publication history

The pre-publication history for this paper can be accessed here:



## References

[B1] Sternberg JM (2004). Human African trypanosomiasis: clinical presentation and immune response. Parasite Immunology.

[B2] Van Nieuwenhove S, Betu-Ku-Mesu VK, Diabakana PM, Declercq L, Bilenge CMM (2001). Sleeping sickness resurgence in the DRC: the past decade. Tropical Medicine and International Health.

[B3] Abaru DE (1985). Sleeping sickness in Busoga, Uganda, 1976-1983. Trop Med Parasitol.

[B4] Mathers CD, Ezzati M, Lopez AD (2007). Measuring the burden of neglected tropical diseases: the global burden of disease framework. Public Library of Science Neglected Tropical Diseases.

[B5] World Health Organization (2006). Human African trypanosomiasis (sleeping sickness): epidemiological update. Weekly Epidemiological Record.

[B6] Jannin JG (2005). Sleeping sickness-a growing problem?. British Medical Journal.

[B7] Simo G, Asonganyi T, Nkinin SW, Njiokou F, Herder S (2006). High prevalence of *Trypanosoma brucei gambiense* group 1 in pigs from the Fontem sleeping sickness focus in Cameroon. Veterinary Parasitology.

[B8] Fèvre EM, Picozzi K, Jannin J, Welburn SC, Maudlin I (2006). Human African trypanosomiasis: epidemiology and control. Advances in Parasitology.

[B9] Fèvre EM, Picozzi K, Fyfe J, Waiswa C, Odiit M, Coleman PG, Welburn SC (2005). A burgeoning epidemic of sleeping sickness in Uganda. Lancet.

[B10] Enyaru JCK, Odiit M, Winyi-Kaboyo R, Sebikali CG, Matovu E, Okitoi D, Olaho-Mukani W (1999). Evidence for the occurrence of *Trypanosoma brucei rhodesiense *sleeping sickness outside the traditional focus in south-eastern Uganda. Annals of Tropical Medicine and Parasitology.

[B11] Picozzi K, Fèvre EM, Odiit M, Carrington M, Eisler M, Maudlin I, Welburn SC (2005). Sleeping sickness in Uganda: a thin line between two fatal diseases. British Medical Journal.

[B12] Fèvre EM, Coleman PG, Odiit M, Magona JW, Welburn SC, Woolhouse MEJ (2001). The origins of a new *Trypanosoma brucei rhodesiense* sleeping sickness outbreak in eastern Uganda. Lancet.

[B13] Okoth JO, Kapaata R (1988). The hosts of *Glossina fuscipes fuscipes* (Newstead) in Busoga, Uganda, and epidemiological implications for trypanosomiasis. Annals of Tropical Medicine and Parasitology.

[B14] Odiit M, Bessell PR, Fèvre EM, Robinson T, Kinoti J, Coleman PG, Welburn SC, McDermott J, Woolhouse MEJ (2006). Using remote sensing and geographic information systems to identify villages at high risk for rhodesiense sleeping sickness in Uganda. Transactions of the Royal Society of Tropical Medicine and Hygiene.

[B15] de la Rocque S, Augusseau X, Guillobez S, Michel V, De Wispelaere G, Bauer R, Cuisance D (2001). The changing distribution of two riverine tsetse flies over 15 years in an increasingly cultivated area of Burkina Faso. Bulletin of Entomological Research.

[B16] Robinson T, Rogers D, Williams B (1997). Mapping tsetse habitat suitability in the common fly belt of Southern Africa using multivariate analysis of climate and remotely sensed vegetation data. Medical and Veterinary Entomology.

[B17] Odiit M, McDermott JJ, Coleman PG, Fèvre EM, Welburn SC, Woolhouse MEJ (2004). Spatial and temporal risk factors for the early detection of *T. b. rhodesiense *sleeping sickness patients in Tororo and Busia districts, Uganda. Transactions of the Royal Society of Tropical Medicine and Hygiene.

[B18] ESRI Scripts Database. http://arcscripts.esri.com.

[B19] Odiit M, Coleman PG, Liu WC, McDermott J, Fèvre EM, Welburn SC, Woolhouse MEJ (2005). Quantifying the level of under-detection of *Trypanosoma brucei rhodesiense *sleeping sickness cases. Tropical Medicine and International Health.

[B20] Wyatt GB, Boatin BA, Wurapa FK (1985). Risk factors associated with the acquisition of sleeping sickness in north-east Zambia; a case-control study. Ann Trop Med Parasitol.

[B21] Robertson DHH (1963). Human trypanosomiasis in south-east Uganda: a further study if the epidemiology of the disease among fishermen an d peasant cultivators. Bulletin of the World Health Organization.

[B22] Okia M, Mbulamberi DB, de Muynck A (1994). Risk factors assessment for *T.b. rhodesiense* sleeping sickness acquisition in S.E. Uganda: a case control study. Ann Soc Belg Med Trop.

[B23] Khonde N, Pepin J, Niyonsenga T, De Wals P (1997). Familial aggregation of *Trypanosoma brucei gambiense* trypanosomiasis in a very high incidence community in Zaire. TTrans R Soc Trop Med Hyg.

[B24] Rogers D (1977). Study of a natural population of *Glossina fuscipes fuscipes* Newstead and a model of fly movement. Journal of Animal Ecology.

[B25] Garcia A, Courtin D, Solano P, Koffi M, Jamonneau V (2006). Human African trypanosomiasis: connecting parasite and host genetics. Trends in Parasitology.

[B26] Waiswa C, Olaho-Mukani W, Katunguka-Rwakishaya E (2003). Domestic animals as reservoirs for sleeping sickness in three endemic foci in south–eastern Uganda. Annals of Tropical Medicine and Parasitology.

[B27] Picozzi K, Tilley A, Fèvre EM, Coleman PG, Magona JW, Odiit M, Eisler MC, Welburn SC (2002). The diagnosis of trypanosome infections: applications of novel technology for reducing disease risk. African Journal of Biotechnology.

[B28] Welburn SC, Picozzi K, Fèvre EM, Coleman PG, Odiit M, Carrington M, Maudlin I (2001). Identification of human-infective trypanosomes in animal reservoir of sleeping sickness in Uganda by means of serum-resistance-associated (SRA) gene. Lancet.

[B29] Okoth JO (1986). Peridomestic breeding sites of *Glossina fuscipes fuscipes* Newst. in Busoga, Uganda, and epidemiologic implications for trypanosomiasis. Acta Tropica.

[B30] Hendrickx G, Napala A, Dao B, Batawui K, Bastiaensen P, De Deken R, Vermeilen A, Vercruysse J, Slingenbergh JH (1999). The area-wide epidemiology of bovine trypanosomosis and its impact on mixed farming in subhumid West Africa; a case study in Togo. Veterinary Parasitology.

